# The impact of population-level HbA_1c_ screening on reducing diabetes diagnostic delay in middle-aged adults: a UK Biobank analysis

**DOI:** 10.1007/s00125-022-05824-0

**Published:** 2022-11-22

**Authors:** Katherine G. Young, Andrew P. McGovern, Inês Barroso, Andrew T. Hattersley, Angus G. Jones, Beverley M. Shields, Nicholas J. Thomas, John M. Dennis

**Affiliations:** 1 Exeter Centre of Excellence in Diabetes (EXCEED), University of Exeter Medical School, Exeter, UK; 2 Department of Diabetes and Endocrinology, Royal Devon and Exeter NHS Foundation Trust, Exeter, UK

**Keywords:** Diabetes, HbA_1c_, Public health, Screening, UK Biobank

## Abstract

**Aims/hypothesis:**

Screening programmes can detect cases of undiagnosed diabetes earlier than symptomatic or incidental diagnosis. However, the improvement in time to diagnosis achieved by screening programmes compared with routine clinical care is unclear. We aimed to use the UK Biobank population-based study to provide the first population-based estimate of the reduction in time to diabetes diagnosis that could be achieved by HbA_1c_-based screening in middle-aged adults.

**Methods:**

We studied UK Biobank participants aged 40–70 years with HbA_1c_ measured at enrolment (but not fed back to participants/clinicians) and linked primary and secondary healthcare data (*n*=179,923) and identified those with a pre-existing diabetes diagnosis (*n*=13,077, 7.3%). Among the remaining participants (*n*=166,846) without a diabetes diagnosis, we used an elevated enrolment HbA_1c_ level (≥48 mmol/mol [≥6.5%]) to identify those with undiagnosed diabetes. For this group, we used Kaplan–Meier analysis to assess the time between enrolment HbA_1c_ measurement and subsequent clinical diabetes diagnosis up to 10 years, and Cox regression to identify clinical factors associated with delayed diabetes diagnosis.

**Results:**

In total, 1.0% (1703/166,846) of participants without a diabetes diagnosis had undiagnosed diabetes based on calibrated HbA_1c_ levels at UK Biobank enrolment, with a median HbA_1c_ level of 51.3 mmol/mol (IQR 49.1–57.2) (6.8% [6.6–7.4]). These participants represented an additional 13.0% of diabetes cases in the study population relative to the 13,077 participants with a diabetes diagnosis. The median time to clinical diagnosis for those with undiagnosed diabetes was 2.2 years, with a median HbA_1c_ at clinical diagnosis of 58.2 mmol/mol (IQR 51.0–80.0) (7.5% [6.8–9.5]). Female participants with lower HbA_1c_ and BMI measurements at enrolment experienced the longest delay to clinical diagnosis.

**Conclusions/interpretation:**

Our population-based study shows that HbA_1c_ screening in adults aged 40–70 years can reduce the time to diabetes diagnosis by a median of 2.2 years compared with routine clinical care. The findings support the use of HbA_1c_ screening to reduce the time for which individuals are living with undiagnosed diabetes.

**Graphical abstract:**

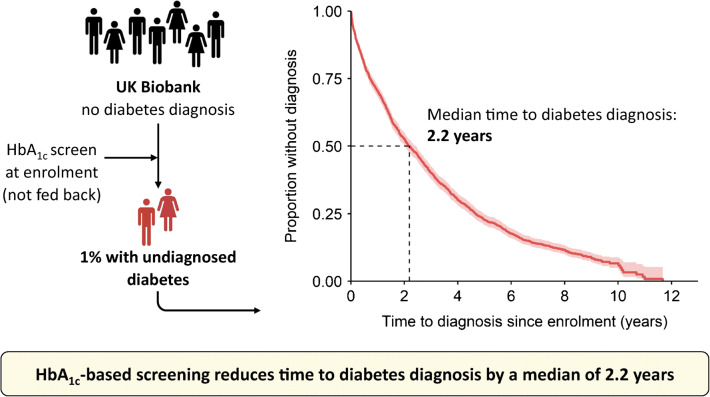

**Supplementary Information:**

The online version contains peer-reviewed but unedited supplementary material available at 10.1007/s00125-022-05824-0.

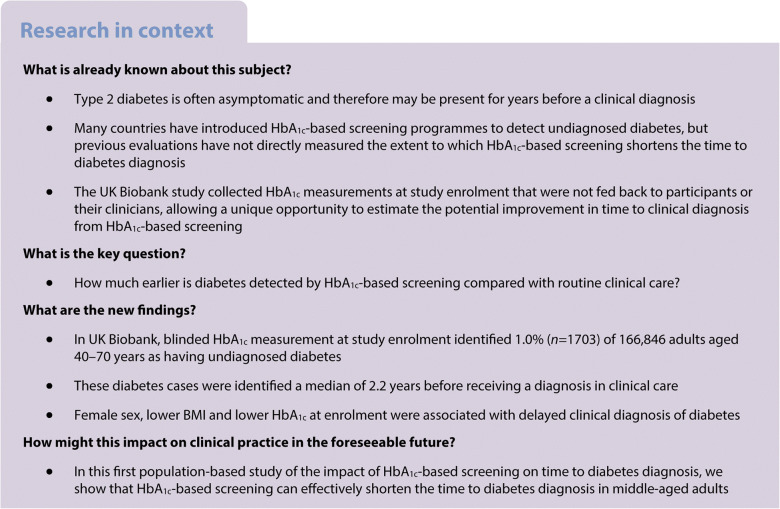



## Introduction

Screening can detect cases of undiagnosed diabetes earlier than symptomatic or incidental diagnosis, allowing for earlier intervention, which may reduce the risk of diabetes complications [1]. Many countries have diabetes screening programmes, with differing eligibility criteria and population coverage. For example, in England, the NHS Health Check, which was initiated in 2009, provides screening for high-risk adults aged 40–74 years [2], whereas in the USA the ADA recommends screening for all adults aged 35 and older [3]. Previous studies have been able to determine the number of cases of undiagnosed diabetes that could be identified by screening programmes [4, 5]. However, as the screening results in these studies directly informed diabetes diagnosis, it was not possible to measure how much earlier diagnosis by screening occurs compared with diagnosis in routine clinical care, and therefore the reduction in the time that people are living with undiagnosed diabetes that could be achieved by screening.

UK Biobank is a population-based cohort of over 500,000 people aged 40–70 years at enrolment [6]. All participants had their HbA_1c_ measured at enrolment, but the results were not reported back to participants or their clinicians. UK Biobank also has linked routine healthcare data providing up to 12 years of follow-up post enrolment. This uniquely allows assessment of the prospective time to diabetes diagnosis in routine care following identification of an elevated HbA_1c_ level (≥48 mmol/mol [≥6.5%]) at enrolment in those without a pre-existing diabetes diagnosis.

We aimed to use UK Biobank data to estimate the reduction in time to diabetes diagnosis that could be achieved by implementing HbA_1c_-based population-level screening, compared with routine care. We also aimed to identify participant characteristics associated with longer time to diagnosis in routine care and assess the performance of widely used selective screening strategies for detecting participants with undiagnosed diabetes.

## Methods

### Study population

**Fig. 1:**
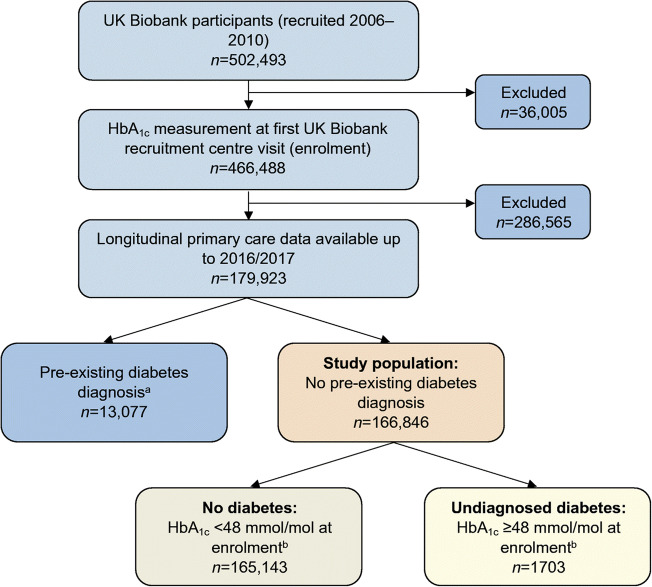
Flow chart of study population inclusion and categorisation into pre-existing diabetes diagnosis, no diabetes or undiagnosed diabetes subgroups. ^a^A pre-existing diabetes diagnosis was defined as a diabetes diagnosis, diabetes-specific complications, diabetes-specific processes of care or evidence of being prescribed glucose-lowering medication, glucagon or glucose testing strips in self-reported or linked primary or secondary healthcare data; or HbA_1c_ ≥48 mmol/mol, fasting glucose ≥7.0 mmol/l or random/2 h postprandial glucose ≥11.1 mmol/l in linked primary healthcare data. ^b^UK Biobank-measured HbA_1c_ values were first calibrated to align with primary care measurements (see Methods, Definitions, Diabetes status)

UK Biobank is a population-based cohort study of 502,493 UK residents aged 40–70 years recruited between 2006 and 2010 [6]. Our study population consisted of 166,846 participants who had their HbA_1c_ level (within the reportable range [7]) measured at UK Biobank enrolment (i.e. the equivalent of an HbA_1c_ screening test). Eligible participants were also required to have linked longitudinal primary care data (up to 2016/2017, ~45% of the UK Biobank cohort) and no pre-existing diabetes diagnosis (*n*=13,077; see Definitions, Diabetes status, and Fig. 1). Linked longitudinal primary care data, defined as a period of continuous primary care registration starting before or on the same day as enrolment and finishing on the same day as or later than the day of enrolment, allowed us to reliably identify incident diabetes diagnoses since UK Biobank enrolment.

Participants with linked primary care data had similar sociodemographic characteristics and enrolment HbA_1c_ levels to those without linked data (see electronic supplementary material [ESM] Table 1).

### Definitions

#### Diabetes status

Participants with a pre-existing diabetes diagnosis were those with any of the following recorded prior to/at enrolment in self-reported or linked primary or secondary healthcare data: a diabetes diagnosis (any type of diabetes including gestational, genetic and secondary diabetes); diabetes-specific complications; diabetes-specific processes of care; or evidence of taking or being prescribed glucose-lowering medication or glucagon or being prescribed glucose testing strips. Participants with an HbA_1c_ level ≥48 mmol/mol (≥6.5%), fasting glucose ≥7.0 mmol/l or random/2 h postprandial glucose ≥11.1 mmol/l recorded in linked primary care data prior to enrolment were also defined as having a pre-existing diabetes diagnosis (see ESM Methods, Definitions, Diabetes status, for details).

In participants without a pre-existing diabetes diagnosis, undiagnosed diabetes was defined as a calibrated HbA_1c_ measurement of ≥48 mmol/mol (≥6.5%) at UK Biobank enrolment. As previously reported, UK Biobank-measured HbA_1c_ levels are systematically lower than UK primary care measurements, so they were calibrated using the following equation: [calibrated HbA_1c_]=0.9696[raw HbA_1c_] + 3.3595 [8]. We also performed sensitivity analysis using uncalibrated HbA_1c_ measurements to define those with undiagnosed diabetes.

#### Baseline characteristics

Participant health information was collected at enrolment (baseline assessment) through a touchscreen questionnaire, a nurse-led interview, biometric measurements and collection of blood, urine and saliva samples. Socioeconomic status was determined using the Index of Multiple Deprivation (IMD). Definitions for all baseline characteristics derived from UK Biobank are reported in ESM Table 2.

#### Time to diabetes diagnosis in routine care

Time to diabetes diagnosis was calculated as the time between UK Biobank enrolment and the estimated date of clinical diabetes diagnosis, defined as the earliest of a code for diabetes, a prescription for glucose-lowering medication, an HbA_1c_ measurement ≥48 mmol/mol (≥6.5%), fasting glucose ≥7.0 mmol/l or random/2 h postprandial glucose ≥11.1 mmol/l in primary care data, or a code indicating diabetes in secondary care data (see ESM Methods, Definitions, Time to diabetes diagnosis in routine care). Participants were censored at the earliest of their date of death (if observed), the date they deregistered from their primary care practice or the end of the longitudinal primary care follow-up available. HbA_1c_ at diagnosis was defined as the closest HbA_1c_ measurement recorded in primary care within 100 days of the estimated date of diagnosis. Median time to diagnosis was calculated for the whole group with undiagnosed diabetes at enrolment. To observe potential changes in time to diagnosis with calendar year of enrolment, we further stratified participants by year of UK Biobank recruitment (2008–2010, when all those with undiagnosed diabetes were enrolled) as a sensitivity analysis.

#### Selective screening strategies

Five strategies for selecting which individuals in a population should be screened for diabetes were investigated for all participants: two based on simple clinical criteria (age ≥60 years, BMI ≥30 kg/m^2^) and three based on validated diabetes risk scores (Leicester Risk Score [LRS] [9], ADA Risk Test score [ADA-RTS] [10] and Finnish Diabetes Risk Score [FINDRISC] [11]). For the three validated risk scores, we used cut-offs suggested by the developers (LRS ≥16 [9], ADA-RTS ≥5 [10], FINDRISC ≥9 [11]), as well as two alternative FINDRISC cut-offs used in previous studies (≥12 [9] and ≥15 [12]). Clinical information required for each strategy was derived from UK Biobank enrolment and linked primary care data (see ESM Table 2).

### Statistical analysis

#### Baseline characteristics

Age, BMI, HbA_1c_ levels and follow-up times are presented as medians with IQRs because of the non-normal distribution of these variables. χ^2^ tests were used to compare categorical variables and unpaired *t* tests were used to compare continuous variables between those with a pre-existing diabetes diagnosis, those with undiagnosed diabetes and those without diabetes.

#### Time to diabetes diagnosis in routine care

Median time to diabetes diagnosis was estimated using the Kaplan–Meier method. A Cox proportional hazards model was fitted to assess associations between age, sex, BMI, HbA_1c_ level, ethnicity, IMD quintile and time to diagnosis, with age, BMI and HbA_1c_ level categorised into clinically relevant categories to aid model interpretability (age: 40–49 years, 50–59 years, 60–70 years; BMI: <30 kg/m^2^, ≥30 kg/m^2^; HbA_1c_: 48.0–52.9 mmol/mol [6.5–7.0%], 53.0–57.9 mmol/mol [7.0–7.4%], ≥58.0 mmol/mol [≥7.5%]). Complete case analysis was used as this excluded <5% of participants. The proportional hazards assumption was violated because of the convergence in survival probability of those in the two highest HbA_1c_ categories (53–57.9 mmol/mol and ≥58 mmol/mol) at approximately 6 years post enrolment (the violation was not prevented by using different HbA_1c_ category cut-offs or treating HbA_1c_ as a continuous variable). However, as most of those in the study population were censored earlier than this, we expect that this had a limited impact on the HRs. A Cox proportional hazards model was also used to assess the association between calendar year of recruitment and time to diagnosis.

#### Evaluation of selective screening strategies

The performances of the five selective screening strategies in selecting those with undiagnosed diabetes were compared with whole population-based HbA_1c_ screening in terms of (1) the number needed to screen (NNS) to find one case of undiagnosed diabetes (1 divided by the proportion of those with undiagnosed diabetes in the population selected to be screened) and (2) the proportion of participants with undiagnosed diabetes not selected for screening (out of those who have all the variables required to implement the screening strategy). Each strategy was assessed overall and in subgroups defined by obesity (BMI ≥30 kg/m^2^).

All analyses were performed using R (version 4.1.1) [13].

## Results

### In total, 1.0% (*n=*1703) of participants without a pre-existing diabetes diagnosis had undiagnosed diabetes at enrolment

Of 166,846 participants without a pre-existing diabetes diagnosis, 1.0% (95% CI 1.0, 1.1; *n*=1703; Fig. 1) had a UK Biobank-measured HbA_1c_ level ≥48 mmol/mol (≥6.5%) indicating undiagnosed diabetes (median [IQR] 51.3 mmol/mol [49.1–57.2] or 6.8% [6.6–7.4]; Table 1). This group with undiagnosed diabetes represents an extra 13.0% (95% CI 12.4, 13.6) of diabetes cases in the study population relative to the 13,077 with known diabetes. Compared with those without diabetes, those with undiagnosed diabetes were on average older, had a higher BMI, were more predominately male, were living in more deprived areas and were more likely to self-report being of non-white ethnicity (all *p*<0.001; Table 1).

**Table 1:** Baseline characteristics of UK Biobank study population at enrolment

Baseline characteristic	(a) Diabetes diagnosis	(b) Undiagnosed diabetes	*p* value (a) vs (b)	(c) No diabetes	*p* value (b) vs (c)
*n*	13,077	1703		165,143	
Age, years	61.4 (55.1–65.6)	60.9 (55.0–65.3)	0.07	58.2 (50.5–63.6)	<0.001
Age category					
40–49 years	1654 (12.6)	225 (13.2)		38,907 (23.6)	
50–59 years	3878 (29.7)	538 (31.6)		55,538 (33.6)	
60–70 years	7545 (57.7)	940 (55.2)		70,698 (42.8)	
Male sex	7587 (58.0)	993 (58.3)	0.84	73,481 (44.5)	<0.001
BMI, kg/m^2a^	30.1 (26.8–34.1)	30.9 (28.1–34.8)	<0.001	26.6 (24.1–29.6)	<0.001
BMI category^a^					
<30 kg/m^2^	6424 (49.5)	699 (41.5)		127,486 (77.4)	
≥30 kg/m^2^	6564 (50.5)	987 (58.5)		37,148 (22.6)	
Non-white ethnicity^a^	1274 (9.8)	192 (11.4)	0.0498	5943 (3.6)	<0.001
IMD quintile^a^			0.73		<0.001
1 (most affluent)	3091 (24.2)	389 (23.3)		52,821 (32.8)	
2	2498 (19.6)	329 (19.7)		37,058 (23.0)	
3	2362 (18.5)	296 (17.7)		29,022 (18.0)	
4	2489 (19.5)	330 (19.8)		24,616 (15.3)	
5 (most deprived)	2335 (18.3)	324 (19.4)		17,600 (10.9)	
HbA_1c_, mmol/mol	48.0 (40.6–57.6)	51.3 (49.1–57.2)		37.3 (35.0–39.5)	
HbA_1c_, %	6.5 (5.9–7.4)	6.8 (6.6–7.4)		5.6 (5.4–5.8)	
HbA_1c_ category					
<48.0 mmol/mol (<6.5%)	6532 (50.0)	0 (0.0)		165,143 (100.0)	
48.0–52.9 mmol/mol (6.5–7.0%)	1930 (14.8)	1050 (61.7)		0 (0.0)	
53.0–57.9 mmol/mol (7.0–7.4%)	1459 (11.2)	264 (15.5)		0 (0.0)	
≥58.0 mmol/mol (≥7.5%)	3156 (24.1)	389 (22.8)		0 (0.0)	

Continuous variables are presented as median (IQR) and categorical variables as *n* (%)

Study population was stratified into (a) evidence of a pre-existing diabetes diagnosis (self-reported diabetes/diabetes-specific complications or processes of care/diabetes medication or any of these in linked healthcare records, or HbA_1c_ ≥48 mmol/mol in primary care records); (b) undiagnosed diabetes (as indicated by a calibrated enrolment HbA_1c_ ≥48 mmol/mol); and (c) no diabetes

^a^Missing data were present for BMI: (a) *n*=89 (0.7%), (b) *n*=17 (1.0%), (c) *n*=509 (0.3%); ethnicity: (a) *n*=78 (0.6%), (b) *n*=12 (0.7%), (c) *n*=574 (0.3%); and IMD quintile: (a) *n*=302 (2.3%), (b) *n*=35 (2.1%), (c) *n*=4026 (2.4%)

Those with undiagnosed diabetes had similar characteristics to those with a pre-existing diagnosis, with no differences in age, sex or deprivation index (all *p*>0.05; Table 1). However, those with undiagnosed diabetes were more likely to self-report being of non-white ethnicity (11.4% vs 9.8%, *p*=0.0498) and had a slightly higher BMI (30.9 vs 30.1 kg/m^2^, *p*<0.001) than those with a pre-existing diagnosis (Table 1).

### Participants with undiagnosed diabetes waited a median of 2.2 years to receive a clinical diagnosis

**Fig. 2:**
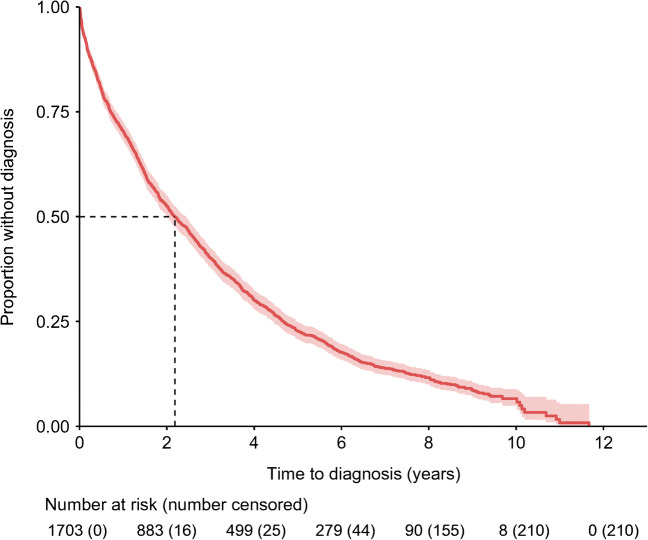
Kaplan–Meier plot of time to diagnosis for those with undiagnosed diabetes at enrolment (*n*=1703). Lighter red shading: 95% CI; dashed black line: median time to diagnosis (2.2 years)

Over a median of 7.3 years’ (IQR 6.7–8.2) follow-up, 87.7% (95% CI 86.1, 89.2; *n*=1493) of the 1703 participants with an HbA_1c_ level ≥48 mmol/mol (≥6.5%) at enrolment subsequently received a clinical diagnosis of diabetes. The median time to receive a clinical diagnosis was 2.2 years (95% CI 2.0, 2.4; Fig. 2). The follow-up period was similar for those receiving and those not receiving a clinical diagnosis (7.1 years [IQR 6.3–8.1] vs 7.4 years [IQR 6.8–8.2], *p*<0.001). Median HbA_1c_ at diagnosis for the 76.3% (*n*=1300) with an at-diagnosis HbA_1c_ level recorded in primary care was 58.2 mmol/mol (IQR 51.0–80.0) (7.5% [IQR 6.8–9.5]). The median increase in HbA_1c_ between UK Biobank enrolment and diagnosis was 4.1 mmol/mol (IQR –0.5–15.4) (0.4% [IQR –0.05–1.4]).

In total, 6.9% (117/1703) of participants with an HbA_1c_ level ≥48 mmol/mol (≥6.5%) at enrolment subsequently had an HbA_1c_ level reported in primary care data below the threshold for diabetes diagnosis (<48 mmol/mol [<6.5%]) at a median time of 4.4 years [IQR 2.4–5.6] after enrolment (this does not include post-diagnosis HbA_1c_ values, which may be altered by lifestyle modification or treatment). A total of 53% of this group (62/117) subsequently received a diabetes diagnosis, a median of 1.2 years (IQR 0.3–2.4) after recording a below-threshold (<48 mmol/mol [<6.5%]) HbA_1c_ level.

Sensitivity analysis using an uncalibrated UK Biobank HbA_1c_ level ≥48 mmol/mol (≥6.5%) to define undiagnosed diabetes identified 1076 individuals with undiagnosed diabetes, with a median time to diagnosis of 1.5 years (95% CI 1.4, 1.7) and with 995 of the 1076 individuals (92.5%, 95% CI 90.9, 94.0) receiving a clinical diagnosis within the study period.

Compared with those enrolled in 2008 (*n*=820), the median time to diagnosis for those enrolled in 2010 (*n*=239) was significantly shorter (1.8 years [95% CI 1.4, 2.2] vs 2.3 years [95% CI 2.1, 2.6], HR [95% CI] 1.22 [1.04, 1.43], *p*=0.017). Those enrolled in 2009 (*n*=644) had a median time to diagnosis of 2.2 years (95% CI 1.9, 2.6), which was not significantly different from the median time to diagnosis for those enrolled in 2008.

### Men and individuals with obesity received a clinical diabetes diagnosis earlier than women and individuals without obesity

For those with undiagnosed diabetes at enrolment, male sex (compared with female sex) and enrolment BMI ≥30 kg/m^2^ (compared with BMI <30 kg/m^2^) were associated with shorter time to diabetes diagnosis (HR [95% CI] for male sex: 1.12 [1.00, 1.25]; HR [95% CI] for BMI ≥30 kg/m^2^: 1.25 [1.12, 1.39]; ESM Table 3). Higher enrolment HbA_1c_ was also strongly associated with shorter time to diagnosis (reference category HbA_1c_ 48.0–52.9 mmol/mol; HR [95% CI] for HbA_1c_ 53.0–57.9 mmol/mol: 2.13 [1.84, 2.46]; HR [95% CI] for HbA_1c_ ≥58.0 mmol/mol: 2.71 [2.37, 3.09]; ESM Table 3). There was no clear evidence of an association between age, ethnic group or socioeconomic status and time to diabetes diagnosis (ESM Table 3).

### Selective screening strategies based on validated risk scores can reduce the number needing to be screened by approximately half but miss 15–30% of undiagnosed diabetes cases

In the study population without a pre-existing diabetes diagnosis (*n*=166,846), the number needed to screen (NNS) to identify one case of undiagnosed diabetes was 98 (95% CI 94, 103). Using simple criteria (age ≥60 years or [separately] BMI ≥30 kg/m^2^) to select which individuals should be screened reduced the NNS to 77 (95% CI 72, 82) and 39 (95% CI 37, 42), respectively, but missed >40% of those with undiagnosed diabetes (Table 2).

**Table 2:** Performance of selective screening strategies compared with population-level screening for identifying those with undiagnosed diabetes

Selective screening strategy	Number needed to screen (NNS) (95% CI)^a^	Percentage of undiagnosed diabetes cases missed (95% CI)^b^
Population level^c^	98 (94, 103)	0.0
Age ≥60 years	77 (72, 82)	44.8 (42.4, 47.2)
BMI ≥30 kg/m^2^	39 (37, 42)	41.5 (39.1, 43.8)
LRS ≥16 (‘high’ to ‘very high’ risk)	48 (45, 50)	15.7 (13.9, 17.4)
ADA-RTS ≥5	51 (48, 53)	23.3 (21.3, 25.4)
FINDRISC ≥9 (includes some of ‘slightly elevated’ category, plus all of ‘moderate’ to ‘very high’ risk)	68 (65, 72)	7.8 (6.4, 9.2)
FINDRISC ≥12 (‘moderate’ to ‘very high’ risk)	49 (46, 52)	30.2 (27.8, 32.5)
FINDRISC ≥15 (‘high’ to ‘very high’ risk)	37 (34, 40)	64.4 (62.0, 66.9)

^a^Of participants selected by the strategy, the number that would need to be screened for diabetes to identify one case of undiagnosed diabetes

^b^Percentage of those with undiagnosed diabetes not selected by the strategy. Only those with complete variables for the selective screening strategy were included

^c^The study population includes those aged 40–70 years only

Selective screening based on the FINDRISC ≥12 strategy gave a similar NNS to those obtained for the LRS ≥16 and ADA-RTS ≥5 strategies (range 48–51; Table 2), with all three strategies giving a NNS that was approximately half that seen for population-level screening. Using these risk score cut-offs, the proportion of missed undiagnosed cases was smaller than for the simple criteria above (range 15.7–30.2%), with the LRS ≥16 strategy missing the least number of cases (Table 2). Using a higher FINDRISC risk score cut-off reduced the NNS further but missed more undiagnosed diabetes cases (FINDRISC ≥15: NNS=37, missed cases=64.4%; Table 2); the converse was true for a lower FINDRISC risk score cut-off.

All risk scores and cut-offs tested performed better in identifying those with undiagnosed diabetes with obesity (BMI ≥30 kg/m^2^), identifying 48–100% of those with undiagnosed diabetes compared with 19–82% in the group without obesity (BMI <30 kg/m^2^; ESM Table 4).

## Discussion

In this UK Biobank population-based cohort of adults aged 40–70 year, 1.0% of those without a pre-existing diabetes diagnosis had undiagnosed diabetes at enrolment. Over a follow-up of up to 12 years, 87.7% of these participants received a clinical diagnosis. Our study demonstrates that HbA_1c_-based screening at UK Biobank enrolment would have identified these diabetes cases a median of 2.2 years before they received a clinical diagnosis. Selective screening strategies based on UK, US and European risk scores were effective overall in targeting those most at risk of undiagnosed diabetes for screening.

This is the first study to use real-world clinical data to determine by how much diabetes diagnoses would be brought forward in a large population cohort by the implementation of a diabetes screening programme. Our finding of a reduction of 2.2 years in time to diagnosis is similar to the reduction of 3.3 years reported in the Ely study (40–65 year olds), which estimated the reduction in time to diagnosis achieved by screening by comparing outcomes for participants randomised to screening at 5-yearly intervals (*n=*92) with outcomes for those receiving no screening (*n=*60) [14]. The shorter reduction in time to diagnosis in this study compared with the Ely study estimate may reflect population differences between the participants, such as the above-average socioeconomic status of UK Biobank participants [15], or our more contemporary study period (2006–2017 in this study vs 1990–2003 in the Ely study). In particular, the 2011 approval by the WHO of HbA_1c_ testing to diagnose diabetes [16], which is easier to perform than the alternative method of fasting glucose testing, led to an increase in opportunistic HbA_1c_ testing [14, 17]. In addition, testing for diabetes may have increased as a result of the introduction of the NHS Health Check in England in 2009 (although, as take-up was initially low, most Health Checks of UK Biobank participants are likely to have occurred after their study period [18]). The effect of these changes in clinical practice may explain the reduction in time to diagnosis seen in our study between recruitment in 2008 and recruitment in 2010 (2.3 years vs 1.8 years).

We found a prevalence of undiagnosed diabetes (in those without known diabetes) of 1.0%, similar to the 1.4% estimate derived from modelling approaches by Diabetes UK [19]. This result was corroborated by a 2003–2005 UK National Screening Committee (NSC) pilot programme, which also showed a prevalence of 1.4% [20]. The prevalence of undiagnosed diabetes in older populations has previously been shown to be higher: the same UK NSC study found a prevalence of 2.8% (of *n*=11,449) in those aged over 40 years, while the prevalence in the Ely study (40–65 year olds) was 4.5% (of *n*=1122) [4]. In addition to the higher socioeconomic status of UK Biobank participants compared with the general population, the lower prevalence in our study (40–70 year olds) may reflect the use of HbA_1c_ ≥48 mmol/mol (≥6.5%) to identify undiagnosed diabetes, as opposed to the glucose-based measures used by the UK NSC and Ely studies (fasting plasma glucose ≥7.0 mmol/l or OGTT 2 h blood glucose ≥11.1 mmol/l) [21].

Male sex, higher HbA_1c_ and BMI ≥30 kg/m^2^ at enrolment were associated with shorter time to diagnosis in current clinical practice, suggesting that clinicians are more likely to screen men or individuals with obesity and less likely to screen women or individuals with a BMI <30 kg/m^2^. Our analysis suggests that the modest delay in diagnosis that we observe for women compared with men is not due to differences in age, BMI, enrolment HbA_1c_ or sociodemographic factors (deprivation and ethnicity) between men and women in UK Biobank. There may be other differences between men and women that affect the likelihood of receiving screening that may be particular to the UK Biobank cohort or UK primary care, as previous studies have not found an association between sex and delayed diabetes diagnosis [22, 23]. A potential explanation for the association of higher enrolment HbA_1c_ with shorter time to diagnosis is that participants with higher blood glucose experienced diabetes symptoms sooner, prompting their doctor to test for diabetes.

Our study shows that selective screening strategies were effective overall in targeting those most at risk of undiagnosed diabetes for screening, with the LRS ≥16 strategy missing fewer undiagnosed cases than the FINDRISC ≥12 and ADA-RTS ≥5 strategies for a similar number needed to screen. However, in our study all three scores performed poorly in identifying those with undiagnosed diabetes with a BMI <30 kg/m^2^ compared with those with a BMI ≥30 kg/m^2^, suggesting that there is an opportunity to optimise existing diabetes screening strategies to improve performance in people without obesity.

### Strengths and limitations

A key strength of this study is the systematic baseline HbA_1c_ assessment at UK Biobank enrolment, which was not fed back to participants, providing a unique dataset to evaluate the benefit of HbA_1c_-based screening for time to diabetes diagnosis compared with routine care. While current recommendations are to repeat HbA_1c_ testing to confirm a diabetes diagnosis, especially in asymptomatic patients [16, 24], it was not possible to obtain such data as UK Biobank assessment measures were not repeated post enrolment. In their absence, our long-term follow-up evaluation in routine clinical practice demonstrated that the majority (87.7%) of participants with undiagnosed diabetes at enrolment eventually received a clinical diagnosis, and only 6.9% subsequently had an HbA_1c_ level below the diagnostic threshold for diabetes (<48 mmol/mol [<6.5%]). This supports the validity of our approach to identifying undiagnosed participants using baseline data and single, rather than repeated, calibrated UK Biobank HbA_1c_ values. Even using uncalibrated UK Biobank HbA_1c_ enrolment measures, which are systematically lower than those in UK primary care and so will underestimate the number of participants with an undiagnosed diagnosis [8], there was a clinically significant median time to diagnosis of 1.5 years.

Limitations of our study include that UK Biobank is not a representative UK cohort; previous studies have shown that participants have better health outcomes, are from less deprived areas and are more predominantly of white ethnicity than the general UK population (based on 2011 ethnicity data) [15]. The relatively low numbers of non-white participants may explain the lack of an association between ethnicity and time to diabetes diagnosis and meant that we were unable to examine associations for specific ethnic groups, in particular people of Asian and black ethnicity. Given the known associations between both non-white ethnicity and higher social deprivation and increased risk of diabetes [25], the prevalence of undiagnosed diabetes in this age group in the wider population is likely to be higher than that observed in UK Biobank (1.0%); studies in other UK cohorts give estimates of 2.8–4.5% [4, 20]. In addition, as UK Biobank participants are likely to be more health-conscious than average, similar to volunteers in other research studies [15], they may have more frequent healthcare appointments and so may be diagnosed with diabetes earlier. This suggests that screening initiatives could identify more cases of undiagnosed diabetes and reduce the time to diagnosis even more than the 2.2 years seen in this study. Conversely, increases in opportunistic diabetes testing during and after the study period may mean that any screening initiative implemented today would provide less substantial benefits than the 2.2 year improvement observed in this study, as supported by the reduction in time to diagnosis seen in our study between recruitment in 2008 and recruitment in 2010. Finally, most of the cohort had not accrued sufficient follow-up data to reliably evaluate the impact of delayed diagnosis on diabetes complications. The UK Prospective Diabetes Study (UKPDS) showed that early intensive blood glucose control reduces the risk of later complications (the ‘legacy effect’) and so we would expect earlier diagnosis to result in fewer long-term complications [26], although trials of screening interventions have not shown a significant reduction in all-cause mortality [1, 17]. This needs further exploration when more recent UK Biobank-linked primary care data become available.

### Clinical implications

The results of this study support the use of HbA_1c_-based screening for undiagnosed diabetes by demonstrating that HbA_1c_ testing can shorten the time to diabetes diagnosis in middle-aged adults. The UKPDS study showed that such a reduction in time to diagnosis, with consequential earlier control of blood glucose, is likely to reduce diabetes complications. Unless diabetes risk scores are improved (especially in the low BMI group), population-based screening with HbA_1c_ is the only way to reliably identify undiagnosed diabetes. However, the cost-effectiveness of this approach merits further assessment and will vary based on the degree of HbA_1c_ testing in the underlying population.

### Conclusions

Our study provides the first population-based estimate of the impact of HbA_1c_-based screening on reducing the time to diabetes diagnosis. In UK Biobank, 1.0% of those aged 40–70 years had undiagnosed diabetes, and population-level HbA_1c_ screening could have reduced the time to diabetes diagnosis in this group by a median of 2.2 years. Earlier diagnosis would allow earlier intervention with the potential to reduce the risk of diabetes complications, but this requires further evaluation.

## Supplementary information

ESM 1 (PDF 231 kb)

## Data Availability

UK Biobank data are available through a procedure described at http://www.ukbiobank.ac.uk/using-the-resource/.

## Abbreviations

ADA-RTS: ADA Risk Test Score
FINDRISC: Finnish Diabetes Risk Score
IMD: Index of Multiple Deprivation
LRS: Leicester Risk Score
NNS: Number needed to screen
NSC: National Screening Committee
UKPDS: UK Prospective Diabetes Study
