# Correction to: The impact of population-level HbA_1c_ screening on reducing diabetes diagnostic delay in middle-aged adults: a UK Biobank analysis

**DOI:** 10.1007/s00125-023-05933-4

**Published:** 2023-05-22

**Authors:** Katherine G. Young, Andrew P. McGovern, Inês Barroso, Andrew T. Hattersley, Angus G. Jones, Beverley M. Shields, Nicholas J. Thomas, John M. Dennis

**Affiliations:** 1 Exeter Centre of Excellence in Diabetes (EXCEED), University of Exeter Medical School, Exeter, UK; 2 Department of Diabetes and Endocrinology, Royal Devon and Exeter NHS Foundation Trust, Exeter, UK

**Correction to: Diabetologia**

10.1007/s00125-022-05824-0

The duration of follow-up of the UK Biobank participants was erroneously given as 10 years instead of 12 years in the text. The original article has been corrected.

