# Supplementary material for: The impact of population-level HbA1c screening on reducing diabetes diagnostic delay in middle-aged adults: a UK Biobank analysis
Source: Diabetologia. 2022 Nov 22;66(2):300–9. doi: 10.1007/s00125-022-05824-0 (PMC9807472; doi:10.1007/s00125-022-05824-0)
Supplement: Supplementary file 1 — (PDF 231 kb) [file 125_2022_5824_MOESM1_ESM.pdf]

Electronic Supplementary Material

ESM METHODS

Definitions

Diabetes status

UK Biobank variables and codelists used to indicate a pre-existing diabetes diagnosis at UK Biobank enrolment can be found at https://github.com/drkgyoung/UK_Biobank_codelists/blob/main/enrolment_diabetes_definition.txt

Time to diabetes diagnosis in routine care

See note on codelists below.

Codelists

Codes and lists of names used with UK Biobank baseline assessment (enrolment) variables 20002 (non-cancer illness code, self-reported) and 20003 (current medications, self-reported), as well as linked primary and secondary healthcare data, and guidance on their implementation are available from the following Github repository: https://github.com/drkgyoung/UK_Biobank_codelists.

ESM TABLES

ESM Table 1

Sociodemographic characteristics of those with and without linked longitudinal primary care data in UK Biobank

**Characteristic**	**Without linked primary care data**	**With linked primary care data**
n	310,612	191,881
Age, years	58.1 (50.4-63.6)	58.5 (50.8-63.8)
Male sex	141,877 (45.7%)	87,238 (45.5%)
BMI, kg/m^2 [a]	26.7 (24.1-29.9)	26.8 (24.2-30.0)
Non-white ethnicity [a]	18,622 (6.0%)	8,411 (4.4%)
IMD quintile [a]		
1 (most affluent)	94,144 (31.1%)	59,636 (31.9%)
2	67,619 (22.3%)	42,386 (22.6%)
3	51,948 (17.2%)	33,812 (18.1%)
4	48,644 (16.1%)	29,431 (15.7%)
5 (most deprived)	40,208 (13.3%)	21,929 (11.7%)
HbA_{1c}, mmol/mol [a]	37.5 (35.2-40.1)	37.5 (35.2-40.1)
HbA_{1c}, % [a]	5.6 (5.4-5.8)	5.6 (5.4-5.8)

Continuous variables are presented as median (IQR) and categorical variables as n (%).

[a] Missing data were present for BMI: a) n=1,826 (0.6%), b) n=1,023 (0.5%); ethnicity: a) n=1,929 (0.6%), b) n=847 (0.4%); IMD quintile: a) n=8,049 (2.6%), b) n=4,687 (2.4%); and HbA_{1c}: a) n=24,046 (7.7%), b) n=11,954 (6.3%).

IMD, Index of Multiple Deprivation

ESM Table 2

Derivation of baseline characteristics from UK Biobank variables. All are from first assessment centre visit, except for linked healthcare data used for 'Previous or current blood pressure medication'.

**Baseline characteristic**	**UK Biobank field(s)**	**Additional processing of UK Biobank data**	**Selective screening strategies which use this variable**
Age	34 Year of birth<br>52 Month of birth<br>53 Date of attending assessment centre	15th of month of birth used as date of birth. Age calculated from date of birth and date of first assessment centre visit.	Age ≥60 years, LRS, ADA-RTS, FINDRISC
Sex	31 Sex	N/A	LRS, ADA-RTS
BMI	21001 BMI (body size measures category)<br>23104 BMI (body composition by impedance category)	23104 used where 21001 missing.	BMI ≥30 kg/m^2, LRS, ADA-RTS, FINDRISC
Ethnicity	21000 (Self-reported) ethnic background	Top level groupings used, and then collapsed into 'white' and 'non-white' (included 'Other ethnic group') groups due to small sample size in non-White ethnicities.	LRS
Index of Multiple Deprivation (IMD) quintile	26410 IMD (England)<br>26426 IMD (Wales)<br>26427 IMD (Scotland)	Quintiles were calculated using population IMD distributions for the relevant country and year [1-3] (countries/years of UK Biobank IMD scores can be found in [4]).	
HbA_{1c}	30750 HbA_{1c}	HbA_{1c} values were processed to align them with primary care measurements as per Young at el.[5]	
Family history of diabetes	20107 Illnesses of father<br>20110 Illnesses of mother<br>20111 Illnesses of siblings	If 'diabetes' was recorded in any of these fields, participants were considered to have a family history of diabetes. This was considered missing if all three fields were missing.	LRS, ADA-RTS
Waist circumference	48 Waist circumference	N/A	LRS, FINDRISC
Hypertension	6150 Vascular/heart problems diagnosed by doctor<br>6153 Medication for cholesterol, blood pressure, diabetes, or take exogenous hormones<br>6177 Medication for cholesterol, blood pressure or diabetes	Any of the following indicated hypertension:<br>• 6150: 4 High blood pressure<br>• 6153: 2 Blood pressure medication<br>• 6177: 2 Blood pressure medication<br>• 20002: Any hypertension code [a]<br>• 20003: Any blood pressure medication code [a]	LRS, ADA-RTS

	20002 Non-cancer illness code, self-reported<br>20003 Current medications, self-reported	This was considered missing if 6150, 6153 and 6177 were all missing, and fields 135 and 136 (number of non-cancer illnesses in field 20002 / medications in field 20003) were also both missing.	
Physical inactivity	864 Number of days/week walked 10+ minutes<br>874 Duration of walks<br>884 Number of days/week of moderate physical activity 10+ minutes<br>894 Duration of moderate physical activity<br>904 Number of days/week of vigorous physical activity 10+ minutes<br>914 Duration of vigorous physical activity	Participants were considered 'habitually inactive' (used in ADA-RTS) if they answered '0 [days per week]' to 864, 884 and 904, or if they answered '-2 Unable to walk' to 864 (in which case they were not asked 884 or 894). This was considered missing if 864, 884 and 904 were all missing.<br><br>Participants were defined as having 30 minutes of physical activity daily (used in FINDRISC) if they had ≥210 minutes of physical activity per week (total physical activity = [864*874] + [884*894] + [904*914]). This was considered missing if 864, 884 and 904 were all missing, or if 874, 894 and 914 were all missing.	ADA-RTS, FINDRISC
Daily fruit/vegetable consumption	1289 Cooked vegetable intake<br>1299 Salad / raw vegetable intake<br>1309 Fresh fruit intake<br>1319 Dried fruit intake<br>All tablespoons/days	Participants were defined as consuming fruit/vegetables daily if any of these fields were >0. This was missing if all of these fields were missing.	FINDRISC
Previous/current blood pressure medication	20003 Current medications, self-reported<br>Linked primary care data	Any blood pressure medication code in 20003 [a], or at least two primary care blood pressure medication prescriptions [a] with issue dates ≤ assessment centre date	FINDRISC

Participant answers of 'do not know' and 'prefer not to answer' were treated as missing.

Note that ADA-RTS additionally includes 'history of gestational diabetes mellitus (GDM)' not used here; we defined individuals with previous GDM as having a 'pre-existing diabetes diagnosis' and so excluded them from our study population as this group are generally subject to different diabetes screening guidelines than the general population[6]. FINDRISC additionally includes 'history of hyperglycaemia'; individuals with this were also defined as having 'pre-existing diabetes diagnosis' and so excluded from our study population.

[a] See ESM Methods: Codelists for codes/names used

ADA-RTS, American Diabetes Association Risk Test Score; FINDRISC, Finnish Diabetes Risk Score; LRS, Leicester Risk Score

ESM Table 3

Association of baseline characteristics with time to diabetes diagnosis from a multivariable Cox proportional hazards model (n=1,642 with 1,437 events). Higher hazard ratio associated with a shorter time to diagnosis.

**Baseline characteristic**	**n (%)**	**HR (95% CI)**	**p value**
Age			
40-49 years [a]	225 (13.2%)	1	
50-59 years	538 (31.6%)	1.05 (0.89, 1.25)	0.56
60-70 years	940 (55.2%)	1.06 (0.90, 1.25)	0.48
Male sex	993 (58.3%)	1.12 (1.00, 1.25)	0.045
BMI			
<30 kg/m^2 [a]	699 (41.5%)	1	
≥30 kg/m^2	987 (58.5%)	1.25 (1.12, 1.39)	<0.001
Non-white ethnicity	192 (11.4%)	1.08 (0.91, 1.27)	0.39
IMD quintile			
1 (most affluent) [a]	389 (23.3%)	1	
2	329 (19.7%)	0.99 (0.85, 1.16)	0.93
3	296 (17.7%)	1.22 (1.04, 1.44)	0.017
4	330 (19.8%)	1.08 (0.92, 1.27)	0.34
5 (most deprived)	324 (19.4%)	1.17 (1.00, 1.38)	0.056
HbA_{1c}			
48-52.9 mmol/mol (6.5-7.0%) [a]	1,050 (61.7%)	1	
53-57.9 mmol/mol (7.0-7.4%)	264 (15.5%)	2.13 (1.84, 2.46)	<0.001
≥58 mmol/mol (≥7.5%)	389 (22.8%)	2.71 (2.37, 3.09)	<0.001

61 participants not included in model due to missing BMI, self-reported ethnicity and/or IMD quintile.

[a] Reference category

HR, hazard ratio for explanatory variable as compared to reference category; IMD, Index of Multiple Deprivation

ESM Table 4

Performance of selective screening strategies for identifying those with undiagnosed diabetes and BMI <30 kg/m^2 or ≥30 kg/m^2.

**Selective screening strategy**	**Percentage of undiagnosed diabetes cases with BMI <30 kg/m^2 identified (95% CI)** [b]	**Percentage of undiagnosed diabetes cases with BMI ≥30 kg/m^2 identified (95% CI)** [b]
Population-level [a]	100.0%	100.0%
Age ≥60 years	60.5% (56.9, 64.1)	51.3% (48.1, 54.4)
BMI ≥30 kg/m^2	0%	100.0%
LRS ≥16 ('high' and 'very high' risk)	67.6% (64.1, 71.1)	96.4% (95.2, 97.6)
ADA-RTS ≥5	57.0% (53.2, 60.7)	90.8% (89.0, 92.7)
FINDRISC ≥9 (includes some of 'slightly elevated' category, plus all of 'moderate' to 'very high' risk)	82.2% (79.2, 85.2)	99.5% (99.1, 100.0)
FINDRISC ≥12 ('moderate', 'high' and 'very high' risk)	47.8% (43.9, 51.8)	86.1% (83.8, 88.5)
FINDRISC ≥15 ('high' to 'very high' risk)	19.4% (16.3, 22.5)	47.6% (44.2, 50.9)

[a] Note that study population includes those aged 40-70 years only
[b] Of those with complete variables for the selective screening strategy

ADA-RTS, American Diabetes Association Risk Test Score; FINDRISC, Finnish Diabetes Risk Score; LRS, Leicester Risk Score

References

[1] UK Government (2021) English Indices of Deprivation. https://www.gov.uk/government/collections/english-indices-of-deprivation. Accessed 26 October 2021

[2] Scottish Government (2021) Scottish Index of Multiple Deprivation 2020. https://www.gov.scot/collections/scottish-index-of-multiple-deprivation-2020/. Accessed 26 October 2021

[3] Welsh Government (2021) Welsh Index of Multiple Deprivation. https://gov.wales/welsh-index-multiple-deprivation. Accessed 26 October 2021

[4] UK Biobank (2018) Index of Multiple Deprivation Scores Version 1.0. https://biobank.ctsu.ox.ac.uk/crystal/crystal/docs/imd_baseline.pdf. Accessed 26 October 2021

[5] Young KG, McDonald TJ, Shields BM (2022) Glycated haemoglobin measurements from UK Biobank are different to those in linked primary care records: implications for combining biochemistry data from research studies and routine clinical care. International Journal of Epidemiology 51(3): 1022-1024. https://doi.org/10.1093/ije/dyab265

[6] Vounzoulaki E, Khunti K, Tan B, Davies M, Gillies C (2020) Gestational diabetes: screening uptake, current challenges and the future – a focused review. British Journal of Diabetes 20: 9-14. https://doi.org/10.15277/bjd.2020.236
